# Comparing the Efficacy of a Unique Advanced Bioengineered Type-I Collagen-Based Skin Substitute Versus an Amnion Graft With Standard of Care in the Treatment of Non-healing Diabetic Foot Ulcers: A Randomized Clinical Trial

**DOI:** 10.7759/cureus.78021

**Published:** 2025-01-26

**Authors:** David G Armstrong, Jason Hanft, Maria Surprenant, Adam Isaac, Marissa Carter, Charles Zelen, Subramanian Gunasekaran

**Affiliations:** 1 Surgery, University of Southern California, Los Angeles, USA; 2 Podiatry, Doctors Research Network, Miami, USA; 3 Podiatry, Foot and Ankle Specialists of the Mid-Atlantic (FASMA), Frederick, USA; 4 Statistics, Strategic Solutions, Bozeman, USA; 5 Clinical Studies, Professional Education and Research Institute, Roanoke, USA; 6 Research and Development, Encoll Corporation, Fremont, USA

**Keywords:** amnion, diabetic foot ulcer, graft, placenta, skin substitute, type-1 collagen, wound healing

## Abstract

The increased cost and morbidity associated with diabetic foot ulcers (DFUs) place a substantial strain on the entire global healthcare system. In this trial, 24 subjects with a chronic DFU, Wagner grade 1 (University of Texas grade 1A), were treated with Standard of Care (SOC) therapy and randomized, one-half to receive advanced high-purity Type-I collagen-based skin substitute (HPTC; manufactured by Encoll Corp., Fremont, CA, USA), and the other half to receive a dehydrated human amnion/chorion membrane (dHACM) or viable cryopreserved human placental membrane (vCHPM). The primary study endpoint was percentage wound area reduction (PAR) over the five-week treatment period. Secondary endpoints included healing time, proportion of wounds closed, and mean number of graft applications. By four weeks post-randomization, the mean PAR for the HPTC group was 83.9 versus 71.3 for dHACM or vCHPM. By four weeks, 6/12 (50%) of wounds receiving HPTC healed, compared to 3/12 (25%) in the comparator group (dHACM or vCHPM). There were no adverse events reported in either group. The results of this study suggest that HPTC shows great promise in wound healing in people with DFUs, with the given limitations. We look forward to future studies that will confirm these encouraging results.

## Introduction

Every year, an estimated 18.6 million people around the world develop a diabetic foot ulcer (DFU) [1]. Of the 537 million people living with diabetes worldwide [2], the lifetime incidence of developing a DFU is 34% [3], placing a substantial strain on the entire global healthcare system. In the United States, DFUs account for nearly one-third of the approximately $116 billion in direct costs related to diabetes [4], and precede more than 80% of all lower extremity amputations [5]. For people who experience a DFU, the five-year mortality rate is 30%, and surpasses 70% for those undergoing a major amputation [6].

Although 70% of DFUs have been shown to heal with Standard of Care (SOC) treatments, many progress to chronic wounds that are difficult to heal [7]. Wound healing in people with diabetes is further complicated by infection. Between 50% and 60% of DFUs become infected, with about 20% of moderate to severe infections progressing to amputation [1]. When subjected to consistently high blood glucose levels, changes to the extracellular matrix (ECM) result in decreased collagen deposition and increased matrix metalloproteinases (MMPs) production, both of which inhibit wound healing [8].

Many collagen-containing advanced wound care grafts have been developed to promote healing in chronic wounds, but vary in composition. Type-I collagen provides abundant receptor sites for growth factors such as fibroblasts and is 97% similar across different species. Type-II and Type-III collagen are only 80% similar; thereby, the biocompatibility of such collagen types is significantly lesser than that of Type-I collagen. Furthermore, Type-I collagen lacks the sulfur-containing amino acid cysteine, which constitutes all of the immunogenic proteins, such as immunoglobulins [9], and does not provoke an immune response, making it a favorable wound healing matrix.

High-purity Type-I collagen-based skin substitute (HPTC, manufactured by Encoll Corp., Fremont, CA, USA) is an uncross-linked, biocompatible skin substitute. HPTC is biocompatible and non-immunogenic due to its high-purity Type-I collagen. It is free of contaminants, retaining the native surface chemistry of the Type-I collagen to interact with the underlying cells. Additional phosphorylation of the pure Type-I collagen enhances tissue repair by triggering cell signaling pathways involved in wound healing. Many other collagen grafts are not truly biocompatible due to the presence of immunogenic molecules, such as Type-III collagen, glycosaminoglycans, and elastin.

HPTC is an advanced skin substitute that is FDA-510(k) #K040314 cleared for application on DFUs. It has been shown in case studies and clinical practice to assist in wound healing. Based on these promising findings, a larger trial is needed to further validate the likelihood of wound healing with a weekly or as-needed application of the graft.

## Materials and methods

**Figure 1:**
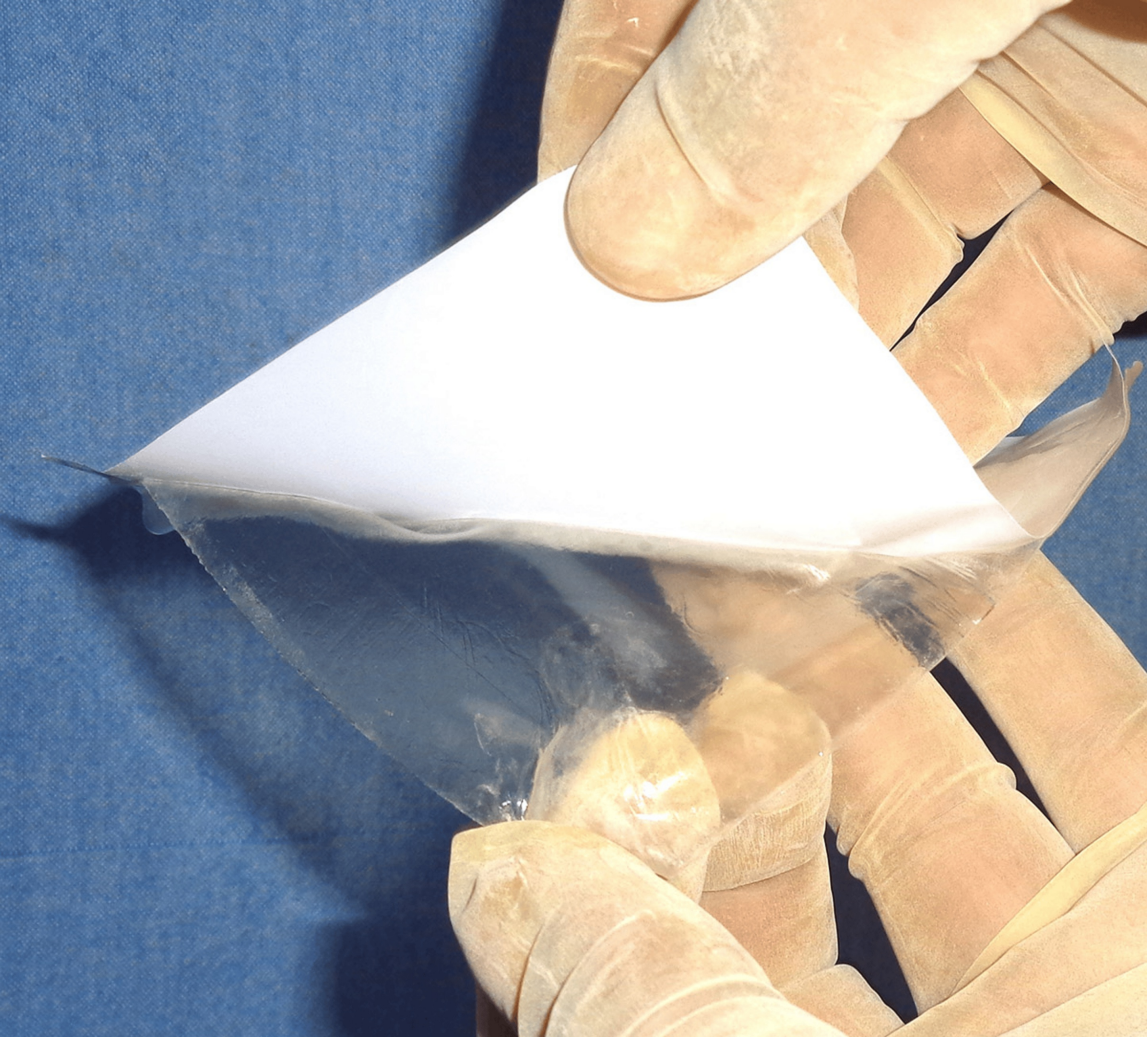
Helicoll advanced Type-I collagen-based skin substitute (Encoll Corp., Fremont, CA, USA) is an uncross-linked, biocompatible phosphorylated Type-I collagen

For this prospective, multi-center, randomized comparative clinical trial, patient outcome data on a commercially available, 510(k) FDA-cleared advanced skin substitute (Figure 1), HPTC, were collected and analyzed. The trial was pre-registered on ClinicalTrials.gov (NCT06557122). The Institutional Review Board of Advarra issued approval under 00000971. Subjects in this trial with a history of a chronic DFU, Wagner grade 1, or subjects with Wagner grade 1 chronic DFUs located on the foot, with at least 50% of the ulcer below the malleolus, measuring between 1 cm² and 20 cm², and present for at least four weeks but no longer than 52 weeks, were eligible for inclusion in the trial. Exclusion criteria included patients with an HbA1c of 13% or higher, measured at or within three months of the initial screening visit, serum creatinine levels ≥3.0 mg/dL within six months of screening, and ulcers exhibiting signs of infection, osteomyelitis, exposed bone, or probing to bone or joint capsule. Table 1 lists the inclusion and exclusion criteria for the trial.

**Table 1:** Inclusion and exclusion criteria

Inclusion criteria	Exclusion criteria
Subjects must be at least 18 years of age or older.	Subjects with a life expectancy of less than 6 months.
Subjects must have a diagnosis of type 1 or 2 diabetes mellitus.	If the target ulcer is infected or has surrounding cellulitis.
At the time of randomization, subjects must have a target diabetic foot ulcer with a minimum surface area of 1.0 cm² and a maximum surface area of 10.0 cm², as measured after debridement using a ruler.	Presence of osteomyelitis or exposed bone, or evidence of bone or joint capsule involvement based on investigator’s examination or radiographic findings.
The target ulcer must have been present for at least 4 weeks and no longer than 52 weeks of standard care prior to the initial screening visit.	Subjects with a target ulcer infection require systemic antibiotic therapy.
The target ulcer must be located on the foot with at least 50% of the ulcer below the malleolus.	Subjects currently receiving immunosuppressive therapy, including systemic corticosteroids at doses exceeding 10 mg/day of prednisone (or equivalent) or cytotoxic chemotherapy.
The target ulcer must be full thickness on the foot or ankle that does not probe to the bone.	Topical steroid application to the ulcer surface within one month prior to initial screening.
Adequate circulation to the affected foot, as documented by one of the following methods performed within 3 months of the first screening visit: (1) Transcutaneous Oxygen Pressure (TCOM) ≥30 mmHg; (2) Ankle-Brachial Index (ABI) between 0.7 and 1.3; (3) Pulse Volume Recording (PVR): Biphasic; (4) Toe-Brachial Index (TBI) ˃0.6; (5) Alternatively, an arterial Doppler ultrasound may be performed to assess the biphasic flow in the dorsalis pedis and posterior tibial vessels at the level of the ankle on the target extremity.	Subjects with a previous partial amputation of the affected foot, where the resulting deformity would interfere with proper offloading of the target ulcer.
If the subject has two or more ulcers, they must be located at least 2 cm apart. The largest ulcer that meets the inclusion and exclusion criteria will be selected as the target ulcer.	A glycated hemoglobin (HbA1c) level of 13% or higher, measured at or within 3 months prior to the initial screening visit.
The subject must consent to use the prescribed off-loading method for the duration of the study.	Serum creatinine levels ≥3.0 mg/dL within the 6 months prior to the initial screening visit.
The subject must agree to attend the twice-weekly/weekly study visits required by the protocol.	Subjects with an acute or inactive Charcot foot that impedes proper offloading of the target ulcer.
The subject must be willing and able to participate in the informed consent process.	Women who are pregnant or planning to become pregnant within the next 6 months.
Patients must read and sign the IRB-approved informed consent form (ICF) prior to the commencement of any screening procedures.	Subjects with end-stage renal disease requiring dialysis.
-	Subjects who have participated in a clinical trial involving an investigational product within the 30 days prior to the screening visit.
-	Subjects with any medical or psychological condition, in the opinion of the Investigator, that could interfere with study assessments.
-	A subject treated with hyperbaric oxygen therapy or a Cellular and/or Tissue Product (CTP) in the 30 days prior to the initial screening visit.
-	A subject who has a sensitivity to bovine (cattle) or ovine (sheep) material.
-	A subject that is allergic to aminoglycoside antibiotics (gentamycin, tobramycin, etc.).

During the screening phase of the study, a series of screening assessments were performed to determine eligibility for the treatment phase. At screening phase visit 1 (SV1), written informed consent form (ICF) was obtained from the subject, and the study index ulcer was identified by the investigator. Each subject could have only one DFU designated as the index ulcer. For subjects with multiple DFUs, the largest ulcer that met the eligibility criteria was selected. If all eligibility criteria were met at the screening visit, the subject proceeded to the first treatment visit. Eligible subjects completed a four-week treatment phase with a one-week follow-up phase, involving weekly evaluations over five weeks, with additional weekly visits occurring during the second and third weeks.

During the treatment phase, the target DFU was debrided as necessary in accordance with SOC. Those subjects meeting eligibility were randomized to one of two groups: (1) SOC with HPTC primary graft applied weekly, or as needed per investigator discretion; or (2) SOC with dehydrated human amnion/chorion membrane (dHACM) or viable cryopreserved human placental membrane (vCHPM) applied weekly, or as needed per investigator discretion. Subjects were evaluated on a weekly basis, with the exception of weeks 2 and 3, during which the patient was seen twice weekly. Weekly and twice-weekly patient outcome evaluations included the investigator’s assessment of target ulcer healing, ulcer size measurements using a ruler to measure wound area, as well as wound photographs. All procedures required during screening were included as part of treatment visit 1 (TV1) for subjects meeting eligibility criteria.

Subjects were seen weekly (±2 days) until the ulcer was healed or they met protocol criteria to exit the study, with the exception of the second and third weeks of treatment, during which the subject was seen twice. For both groups, the assigned treatment was applied only once during the second and third weeks unless the investigator deemed an additional application necessary. If it was determined that the additional application was not necessary, the additional visit involved a dressing change and wound evaluation. The study chair reviewed all photographs of healed wounds to confirm wound healing.

The primary study endpoint was the percentage wound area reduction (PAR) from TV1 to TV5, measured manually with digital photography. Secondary endpoints included the time required to achieve complete wound closure of the target ulcer by the end of five weeks, the proportion of subjects achieving complete closure during the four-week treatment period, and the average number of repeated applications of the advanced skin substitute needed to achieve wound closure.

Statistics

The per-protocol (PP) population comprised randomized subjects, with analysis conducted according to the treatment received. The following subjects were excluded: (a) subjects randomized but later found to be ineligible for the study because they failed to meet all inclusion and exclusion criteria; (b) subjects who did not complete the study; and (c) subjects with major protocol violations. The safety population comprised randomized patients who received at least one treatment. Missing area data were not imputed for PAR analysis. The outcome of complete wound healing was scored as not healed for the following events: the subject died; the subject had an amputation that affects the index ulcer; the subject was lost to follow-up; the subject was withdrawn from the trial; and the subject withdrew consent.

Study variables are presented as means and standard deviations (±SD) for continuous variables and as medians for non-normally distributed data. Categorical variables are reported as counts and proportions or percentages. Statistical testing between treatment groups at baseline was performed to assess the success of randomization. For categorical variables, Chi-square or Fisher's exact tests were used, while for continuous variables, independent t-tests or Mann-Whitney tests were applied, depending on the normality of the data. The PAR for the index wound at X weeks was calculated using the formula: $\frac{(A_1 - A_{xw})}{A_1} \times 100$, where A1 is the area of the index wound at randomization and Axw is the area at X weeks. All endpoint analyses are presented as summaries, and no hypothesis testing was conducted, as this was a pilot trial with insufficient power for comparative testing between treatment groups.

## Results

Subjects in this trial were enrolled at three outpatient centers, between July and October 2024. There were 24 subjects randomized between the two treatment arms: 12 to HPTC and 12 to dHACM or vCHPM. There were three screen failures (11%). No subjects died or were withdrawn during the trial (all 24 subjects completed the trial), and there were no major protocol deviations. Thus, the PP population analyzed was equivalent to an intention-to-treat (ITT) population.

**Figure 2:**
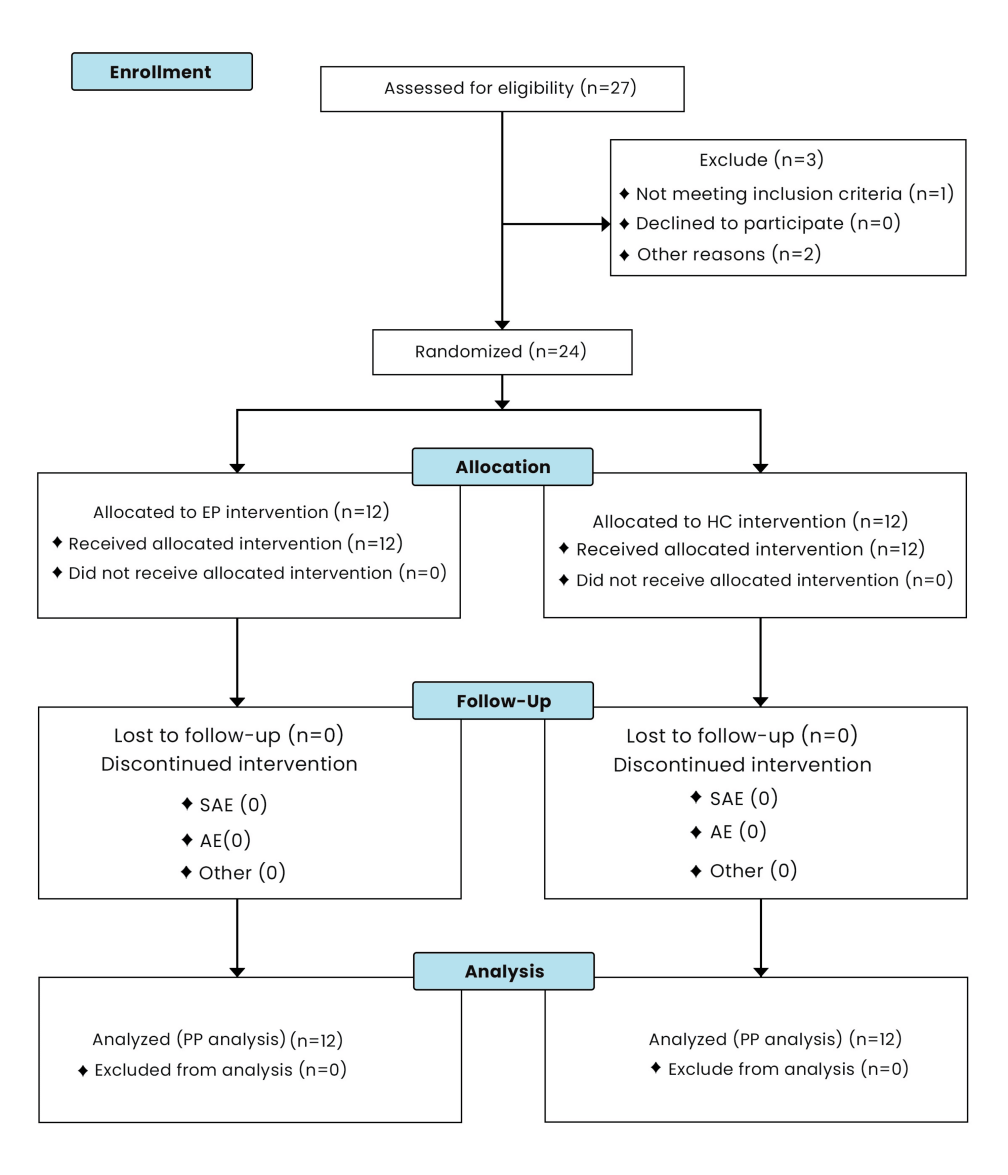
Subject flow chart EP, Comparator Graft (EpiFix or Grafix); HC, Helicoll; SAE, Severe Adverse Event; AE, Adverse Event; PP, Per-Protocol Population

A subject flow chart is presented in Figure 2. A comparison of key subject-related variables between treatment groups is provided in Table 2. The mean age of subjects was 60.8 ± 12.16 years, with 75% male and 91.7% Caucasian subjects. Given the relatively small sample size, the variables were well-balanced between the groups. Wound-related variables are detailed in Table 3 and were well-balanced between groups. The mean baseline wound area was 2.35 ± 1.95 cm². A comparison by treatment group for subject comorbidities (Table 4) showed that variables were well balanced between groups, given the small numbers, except for hypertension, which was substantially different between treatments.

**Table 2:** Comparison by treatment group for key subject-related variables excluding subject comorbidities. Continuous variables are reported as means ± SD, with median/IQR additionally reported for key non-normally distributed continuous variables, and categorical variables as counts (percentage). For categorical variables, Chi-square or Fisher's exact tests were used, while for continuous variables, independent t-tests or Mann-Whitney tests were applied, depending on the normality of the data. DFU: Diabetic Foot Ulcer

Variable	Comparator Graft (n = 12)	Helicoll (n = 12)	p-value
Age (years)	61.1 ± 12.29	60.4 ± 12.03	0.98
Race:			0.34
Caucasian subjects	12 (100)	10 (83)	
African American subjects	0 (0)	1 (8.5)	
Hispanic subjects	0 (0)	1 (8.5)	
Ethnicity: Hispanic subjects	5 (42)	6 (50)	1
Sex at birth:			0.64
Male	10 (83)	8 (67)	
Female	2 (17)	4 (33)	
BMI	29.8 ± 10.61	32.5 ± 5.24	0.44
Smoker:			0.5
Current	1 (8)	0 (0)	
Former	5 (42)	4 (33)	
Never smoked	6 (50)	8 (67)	
HbA1c (%)	7.6 ± 1.69	7.5 ± 2.38	0.95
Creatinine (mg/dL)	0.93 ± 0.35	1.08 ± 0.24	0.22
Years of DFUs	4.9 ± 16.45	5.9 ± 12.98	0.87
Prior DFU count	3.9 ± 8.25; median: 2, IQR: 1	3.8 ± 4.86; median: 2; IQR: 3.3	0.35
Other concurrent DFUs (at screening):			0.3
0	10 (83)	9 (75)	
1	0 (0)	2 (17)	
2	2 (17)	1 (8)	
History DFU recurrence	4 (33)	6 (50)	0.68
Amputations, minor:			0.23
0	6 (50)	9 (76)	
1	1 (8)	1 (8)	
2	5 (42)	1 (8)	
5	0 (0)	1 (8)	
Major amputations	1 (8)	0 (0)	1
Foot deformities:			0.51
Charcot (stable)	2 (17)	1 (8)	
Plantar arthrodesis	1 (8)	0 (0)	
Ankle tarsal tunnel	0 (0)	1 (8)	

**Table 3:** Comparison by treatment group for key wound-related variables ¹At randomization; ²At and post-randomization; NOTE: all subjects used CAM boots in the study. Continuous variables are reported as mean ± SD, with median/IQR additionally reported for key non-normally distributed continuous variables, and categorical variables as counts (percentage). For categorical variables, Chi-square or Fisher's exact tests were used, while for continuous variables, independent t-tests or Mann-Whitney tests were applied, depending on the normality of the data. DFU, Diabetic Foot Ulcer; CAM, Controlled Ankle Motion

Variable	Comparator graft (n = 12)	Helicoll (n = 12)	p-value
Wound area (cm^2^)¹	2.4 ± 1.77; median: 1.6, IQR: 2.4	2.3 ± 2.13; median: 1.6, IQR: 0.9	0.66
Wound age (weeks)¹	23.8 ± 12.4; median: 22.5, IQR: 17.3	16.5 ± 13.63; median: 14, IQR: 18.8	0.19
Vertical location:			1
Plantar	11 (92)	12 (100)	
Dorsal	1 (8)	0 (0)	
DFU position:			1
Medial	7 (58)	8 (67)	
Lateral	5 (42)	4 (33)	
Anatomical location:			0.39
Toe	4 {34)	3 (25)	
Forefoot	6 (50)	5 (42)	
Midfoot	1 (8)	4 (33)	
Heel	1 (8)	0 (0)	
History offloading type:			0.51
No offloading	3 (25)	1 (8)	
CAM boot	5 (42)	7 (58)	
Surgical shoe	4 (33)	4 (34)	
Number of sharp debridements²	3.8 ± 0.94	3.5 ± 1.45	0.63

**Table 4:** Comparison by treatment group for selected subject comorbidities and total count of comorbidities ¹Restricted mobility: any subject who uses a walker, wheelchair, crutches, or canes, and/or has an inability to move freely because of a physical or mental disability, handicap, or restriction; ²Based on all identified comorbidities from medical history. CHF, Chronic Heart Failure; CKD, Chronic Kidney Disease; DFU, Diabetic Foot Ulcer; PAD, Peripheral Arterial Disease; PVD, Peripheral Vascular Disease

Variable	Comparator graft (n = 12)	Helicoll (n = 12)	p-value
CKD	1 (8)	0 (0)	1.0
Hypertension	6 (50)	11 (92)	0.069
PAD/PVD	0 (0)	1 (8)	1.0
CHF	0 (0)	2 (17)	0.48
Leg edema	0 (0)	1 (8)	1.0
Restricted mobility¹	0 (0)	0 (0)	1.0
Venous disease	0 (0)	0 (0)	1.0
Peripheral neuropathy	12 (100)	8 (67)	0.093
Any psychiatric condition	3 (25)	2 (17)	1.0
Comorbidity count²	6.9 (4.06)	7.1 (4.06)	0.86

**Figure 3:**
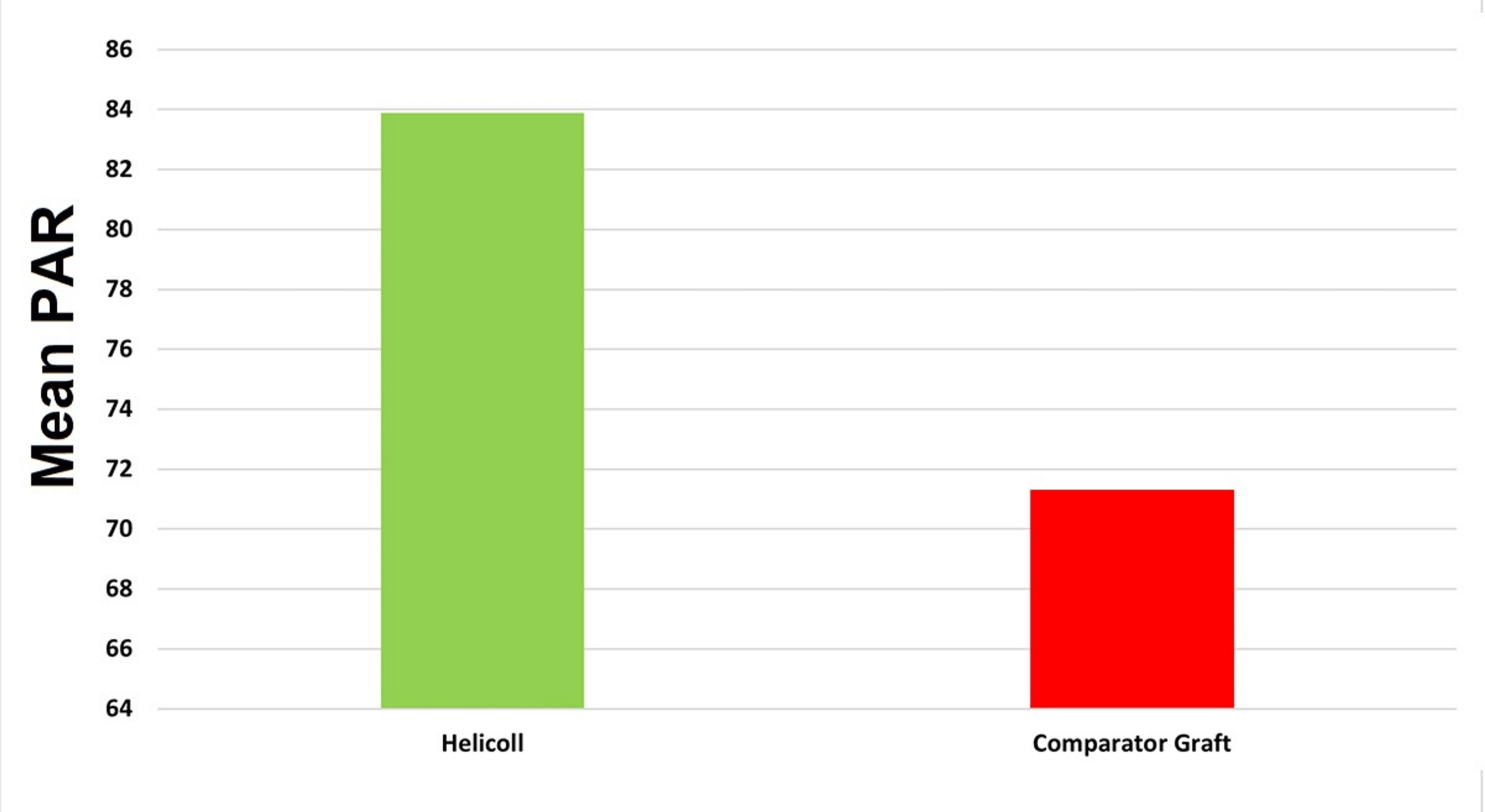
Mean PAR values four weeks post-randomization by treatment group PAR, Percentage Wound Area Reduction

**Figure 4:**
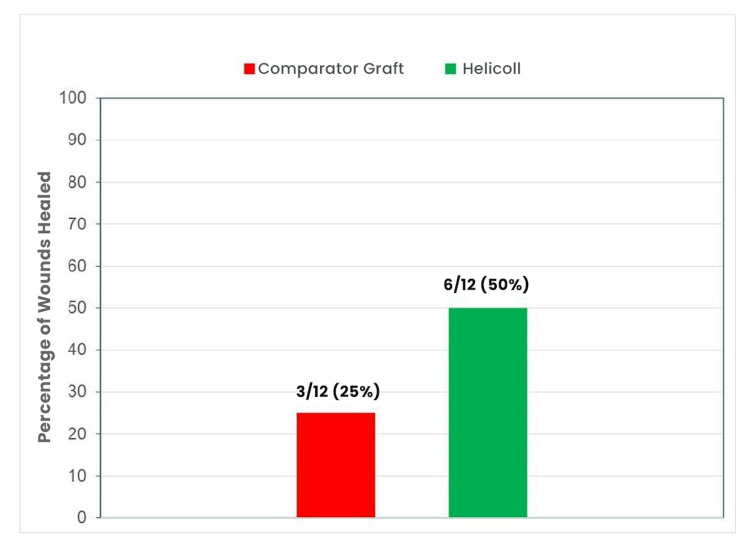
Graphic presentation of the complete wound healing results

**Figure 5:**
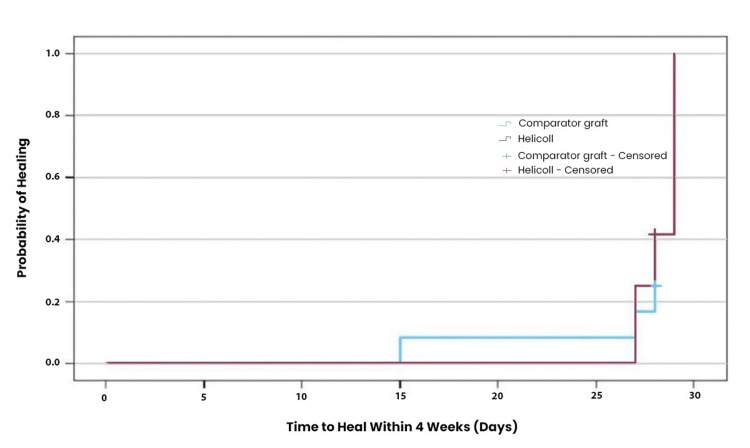
Kaplan-Meier plot at the four-week period shows healing by treatment group

By four weeks post-randomization, the mean PAR for the HPTC group was 83.9, versus 71.3 for the dHACM or vCHPM group (Figure 3). Furthermore, by four weeks, 6/12 (50%) of wounds in the HPTC group healed, compared to 3/12 (25%) in the comparator group (Figure 4). The Kaplan-Meier plot for both treatment groups at the four-week period shows that healing in the HPTC group was approximately double compared to the amnion and placenta group (Figure 5). There were no adverse events reported.

## Discussion

Diabetic foot complications, including chronic ulceration, infection, and limb amputation, continue to rise at a staggering rate. Every 1.2 seconds, a DFU occurs around the world, and every 20 seconds, a limb is amputated [10]. Furthermore, the estimated cost of treating one DFU ranges from $11,700 to $16,883 [11]. Considering the significant morbidity and cost associated with DFUs, the need to develop more effective treatments is essential. No prior clinical studies have directly compared collagen-based skin substitutes with the increasingly marketed amnion-based products for clinical uses. We have completed this study due to the clinical need for such a comparison to evaluate the true effectiveness of two different skin substitutes. This study constitutes a randomized clinical trial to assess and compare the efficacy of HPTC and amnion/placenta-based skin substitutes in the treatment of DFUs.

In this trial, the mean PAR for the HPTC group, by four weeks post-randomization, was 83.9 compared to 71.3 in the dHACM or vCHPM group. Moreover, by four weeks, 50% of wounds in the HPTC group healed, compared to 25% for dHACM or vCHPM. These results compare favorably with previously reported studies and demonstrate the promising potential of the HPTC graft for healing DFUs [12]. These results also align with those presented in a recent study published by Narayan et al. [9], in which subjects with chronic DFUs treated with HPTC achieved improved healing outcomes when compared to the dHACM group.

A key factor contributing to the success of HPTC in these studies may be its composition of high-purity (>97%) Type-I collagen, which is highly biocompatible and preserves the natural properties of collagen. This high purity of Type-I collagen renders the potential biocompatibility. At the same time, the retention of the native structure of collagen, offering 3,000 receptor sites per molecule, supports cell attachment. Keeping the molecules uncross-linked adds to its biological functions toward wound healing. Further, its high interspecies similarity and low immunogenicity give it distinct advantages over other collagen types, making it a highly promising option for advanced wound care applications. One more added feature of HPTC is the phosphorylation of Type-I collagen, which induces bioactivity through cell signaling and enhances its tissue repair and regenerative capabilities. Thus, HPTC integrates seamlessly into the wound, serving as an effective matrix for cellular and vascular regeneration. Its potential mechanism of action is associated with promoting a moist wound environment, facilitating early-stage healing, and supporting neo-vascularization [13,14].

By comparison, human intact tissue membrane-derived products may lose some bioactivity due to chemical cross-linking. Possible local irritation could lead to a rapid cellular response not consistent after three weeks. This could be caused by elastin molecules getting degraded into their monomers, which can have a negative impact, similar to the tissue interactive drawbacks of silicon dioxide-based implantable products [15]. Its cellular activity may be mainly due to its micro-irritability to the cells surrounding the implant, caused by silica (SiO2) [16-18]. This explicitly shows the significance of assessing the safety and efficacy of every component of a bio-matrix, which is usually governed by FDA regulations, before using such a construct for tissue regenerative applications.

Glycosylation is a post-translational modification (PTM) where carbohydrates are attached to proteins. In uncontrolled diabetic patients, excess plasma glucose glycosylates collagen lysine residues, which makes it unsuitable for oxidative deamination of native collagen lysine. As a result, the next steps of aldehyde formation and the normal cross-linking process of collagen through the lysyl oxidase enzyme are inhibited, leading to non-maturation of the wound bed and resulting in an ulcer. This leads to unstable collagen that is susceptible to enzymatic degradation and impaired wound healing. A unique advantage of HPTC in the treatment of DFUs is in reducing the impact of glycosylation. When HPTC is applied directly over the diabetic ulcer wound, it tends to absorb the excess glucose floating in the blood plasma through a common PTM, resulting in the formation of glycosylated collagen. Thereby, following the absorption of a significant amount of glucose floating in the blood serum, the DFU wound collagen would be minimally impacted by the lesser quantity of glucose in the circulating blood, allowing for the maturation of collagen to happen effectively toward the wound healing of the ulcer.

The limitation of the study is the lack of a longer follow-up period after wound healing to assess recurrence rates. Additionally, conducting the study at sites with varied geographical locations is a limitation, though the sponsor plans to extend the study to diverse geographic regions in future research.

## Conclusions

The strengths of our study include a robust trial design with appropriate screening procedures, a standardized approach to SOC treatment, PP analysis, and appropriate adjustments for multiple statistical testing. The results of this study suggest that the Type-I collagen-based skin substitute, HPTC, shows promise in wound healing in people with DFUs. We look forward to future studies that will confirm these initially encouraging results.
